# Development of guidelines to assist organisations to support employees returning to work after an episode of anxiety, depression or a related disorder: a Delphi consensus study with Australian professionals and consumers

**DOI:** 10.1186/1471-244X-12-135

**Published:** 2012-09-03

**Authors:** Nicola J Reavley, Anna Ross, Eoin J Killackey, Anthony F Jorm

**Affiliations:** 1 Orygen Youth Health Research Centre, Centre for Youth Mental Health, University of Melbourne, Locked Bag 10, Parkville, Victoria, 3052, Australia

## Abstract

**Background:**

Mental disorders are a significant cause of disability and loss of workplace productivity. The scientific evidence for how organisations should best support those returning to work after common mental disorders is relatively limited. Therefore a Delphi expert consensus study was carried out with professional and consumer experts.

**Methods:**

A systematic review of websites, books and journal articles was conducted to develop a 387 item survey containing strategies that organisations might use to support those returning to work after common mental disorders. Three panels of Australian experts (66 health professionals, 30 employers and 80 consumers) were recruited and independently rated the items over three rounds, with strategies reaching consensus on importance written into the guidelines.

**Results:**

The participation rate across all three rounds was 60.2% (57.6% health professionals, 76.7% employers, 56.3% consumers). 308 strategies were endorsed as essential or important by at least 80% of all three panels. The endorsed strategies provided information on policy and procedures, the roles of supervisors, employees and colleagues in managing absence and return to work, and provision of mental health information and training.

**Conclusions:**

The guidelines outline strategies for organisations supporting those returning to work after common mental disorders. It is hoped that they may be used to inform policy and practice in a variety of workplaces.

## Background

The 2007 Australian National Survey of Mental Health and Wellbeing estimated that mental disorders affect as many as one in four people aged 16 to 24 in any 12-month period [1]. Depression, anxiety and related disorders (hereafter referred to as common mental disorders) are the most prevalent mental disorders and are among the leading causes of disability worldwide [2]. In addition to social impact, mental disorders can significantly affect workplace productivity due to absenteeism and presenteeism (being unproductive at work) [3,4].

For a great number of people the ability to work is an important aspect of quality of life [5]. Prolonged absence from work is associated with economic and social deprivation and the importance of return to work (RTW) after an episode of depression, anxiety or a related disorder has long been acknowledged [6]. Evidence suggests that impairments in job performance may persist after symptom reduction and efforts are needed to reduce work-impairment secondary to mental health problems [7]. Current rehabilitation and RTW models are usually based on physical conditions and may not be appropriate for those returning after common mental disorders [8,9]. It is only recently that research interest has focused on RTW interventions in those with these disorders [10-12]. Some evidence supports the effectiveness of work-directed interventions in reducing sick leave and improving productivity [13-15].

The literature on the factors facilitating successful RTW after common mental disorders is relatively limited. This is particularly true in the Australian context, as the majority of research has been carried out in Europe and the US, which have different health and occupational health and safety (OHS) regulatory frameworks. Australian employers are legally obliged to support an injured worker’s RTW by obtaining relevant information about the person’s capacity for work, considering reasonable workplace support aids or modifications, proposing options for suitable employment, providing clear and accurate details of the RTW arrangements, and monitoring the RTW process. For a period of 52 weeks, an employer must provide the injured worker with suitable employment if they have an incapacity for work, and/or pre-injury or equivalent work once they have returned to full capacity.

However, implementation of workplace policies and practices is under-researched and remains a significant challenge. While evidence of the effectiveness of interventions may be increasing, workplace health researchers often struggle to effectively communicate research findings to workplace decision-makers. In turn, workplace practices may not adequately inform research. Such knowledge exchange, which incorporates the idea of knowledge as a changing set of understandings shaped by both researchers and users, is increasingly recognised as an effective means of incorporating (or taking up) research information [16]. It involves engaging decision makers in all relevant sectors and represents a move towards viewing practice-based evidence as equally relevant to evidence-based practice [17].

In this context, assessing expert consensus offers a way of bringing together available research evidence and best practice in order to enable recommendations and decisions to be made. Such methods have been widely applied in the development of clinical practice guidelines. The most commonly used consensus method is the Delphi process, which has been used to develop mental health first aid guidelines using the expertise of professionals, consumers and carers [18-20]. These guidelines have been used to revise the content of a Mental Health First Aid training program [21].

IThis paper reports on the development of guidelines for organisations to support those returning to work after common mental disorders. Once established, the guidelines may be used to inform policy and practice in organisations.

## Methods

### The Delphi method

The Delphi process involves a group of experts making private ratings of agreement with a series of statements, feedback to the group of a statistical summary of the ratings, and then another round of rating [22]. Statements about supporting those returning to work after common mental disorders were derived from a search of the lay and scientific literature, and these were presented to a panel of experts in three sequential rounds. Any additional strategies suggested by panel members were included in the subsequent round for all experts to rate. A summary of group ratings was fed back to the panel members after the first two rounds. Panel members could choose to either change or maintain their ratings. In this way, a list of statements that had substantial consensus in ratings was developed, and those statements with low or conflicting ratings discarded.

### Panel formation

There were three separate panels. One comprised professionals in the field, including occupational physicians, psychologists, occupational therapists, mental health consultants and rehabilitation and welfare coordinators. A second panel consisted of consumer advocates, who were asked to participate if they had personal experience of returning to work following an episode of mental illness. The third panel consisted of employers working in the area including human resources professionals, occupational health and safety (OHS) professionals and those in managerial positions.

Health professionals were recruited through the Australian and New Zealand Society of Occupational Medicine (ANZSOM) and the Australasian Faculty of Occupational and Environmental Medicine (AFOEM). Participants were limited to Australian organisations due to differences in health and regulatory systems. Employers were recruited through direct contact from researchers and employer representative organisations (e.g. Chambers of Commerce). Consumers were recruited by distributing information about the study to consumer organizations associated with mental health issues. The study did not aim to get representative samples of experts, because the Delphi method requires panel members who are information and experience rich rather than representative. All those who agreed to be involved were accepted as panel members.

At least 20 members are necessary for each Delphi panel in order to avoid one member having a large influence on the results [22]. In this study, panel membership numbered 176, with 66 health professionals, 30 employers and 80 consumers. 74.9% of panel members were female (69.7% of the professionals, 70.0% of the employers and 80.0% of the consumers). The median age was 46 years for the professionals, 43.5 years for the employers and 43.5 years for the consumers. Of the 66 professionals on the panel, there were 18 occupational physicians, 18 mental health consultants/advisors, 10 psychologists, 8 welfare and rehabilitation coordinators, 4 occupational therapists, 4 mental health researchers, 4 psychiatric nurses and 2 counsellors. Of the 30 employers, there were 13 health and wellbeing advisors/coordinators, 8 human resources personnel and 13 in managerial positions (figures do not add up to 30 due to panel members reporting multiple roles).

### Questionnaire development and administration

A systematic literature review was conducted of websites, books and journal articles for strategies relating to how organisations could support those returning to work after mental health problems (e.g. identifying early signs of relapse, keeping in contact with absent employees). This involved a comprehensive search in Google search engines ( http://www.google.com.au, http://www.google.co.uk, http://www.google.ca, http://www.google.com). The following search terms were entered into each: (depression OR anxiety OR ‘mental disorder’) AND (return-to-work OR ‘return to work’ OR absenteeism OR work resumption OR sickness absence OR relapse prevention OR disability) AND (work OR workplace OR employee). The first 50 sites for each Google search engine were examined for statements about how institutions could support those returning to work after common mental disorders. Any links that appeared on these web pages that the authors thought may contain useful information were followed. Relevant journal articles were located on PsycINFO and PubMed, using the keyword search terms: (depression OR anxiety OR ‘mental disorder’) AND (return-to-work OR ‘return to work’ OR work resumption OR relapse prevention) and the words (work OR workplace OR employee) in the abstract.

We obtained suggestions for how institutions could support those returning to work after common mental disorders from 12 websites (e.g. http://www.guardingmindsatwork.cahttp://www.hse.gov.uk/)*,* 29 journal articles (e.g. [10,23,24] and 40 booklets/factsheets (e.g. [25,26]. The majority of strategies came from the booklets/factsheets. A full list can be obtained from the authors on request. The information gathered from these sources was analysed by one of the authors (AR) and written up as individual survey items. This document was presented to a working group comprising the authors, who screened the items to ensure they fitted the definition of actions that organisations could take to support those returning to work after common mental disorders, were comprehensible, and had a consistent format, while remaining as faithful as possible to the original wording of the information. In addition, the questionnaire content was informed by a small number of strategies suggested by the working group to fill perceived gaps in the questionnaire’s content. After several draft surveys, the group produced a list of 387 items that formed the first survey sent to panel members.

The Round 1 survey was organized into nine sections (see Table 1). Panel members were asked to rate the importance of each item. The rating scale used was: essential, important, depends, unimportant, should not be included, don’t know. The Round 1 survey also included comment boxes that allowed panel members to give feedback after each section. To analyse the comments that panel members had written in the first round questionnaire, one of the authors (AR) read through all the comments and wrote them up as draft strategies. The working group evaluated the suggested draft strategies to determine whether they were original ideas that had not been included in the first round questionnaire. Any strategy that was judged by the group to be an original idea was included as a new item to be rated in the second round questionnaire. Panel members completed the questionnaires online using SurveyMonkey [27]. The study was approved by the Human Research Ethics Committee of the University of Melbourne.

**Table 1:** Round 1 survey sections and number of items

	**Section**	**Number of items**
1.	Policy	30
2.	Organisations	57
3.	Awareness	78
4.	Supervisors	131
5.	The return-to-work plan	27
6.	The employee	40
7.	Employer representative*	64
8.	Colleagues	7
9.	Trade union representatives	7
10.	Friends and family	10

*This section added in Round 2.

### Statistical analysis

On completion of each round, the survey responses were analysed by obtaining percentages for the health professional, employer and consumer panels for each item. The following cut-off points were used:

#### Criteria for accepting an item

• If at least 80% of all panels rated an item as essential or important as a guideline for institutions supporting those returning to work after common mental disorders, it was included in the guidelines.

#### Criteria for re-rating an item

Panel members rerated an item in the next round if:

• 80% or more of the panel members in one group rated an item as essential or important

• 70-79% of panel members in both groups rated an item as either essential or important

#### Criteria for rejecting an item

• Any items that did not meet the above conditions were excluded.

## Results

The participation rate of those who took part in all three rounds was 60.6% (57.6% health professionals, 76.7% employers, 57.0% consumers). See Table 2 for the number of panel members who completed each round.

**Table 2:** Participant numbers for each round of the survey

**Panel**	**Round 1**	**Round 2**	**Round 3**
	**N**	**N (% of round 1)**	**N (% of round 1)**
Consumer	80	57 (71.3)	45 (56.2)
Employer	30	24 (80.0)	23 (76.6)
Professional	66	44 (66.7)	38 (57.6)
Total	176	126 (71.6)	106 (60.2)

**Figure 1:**
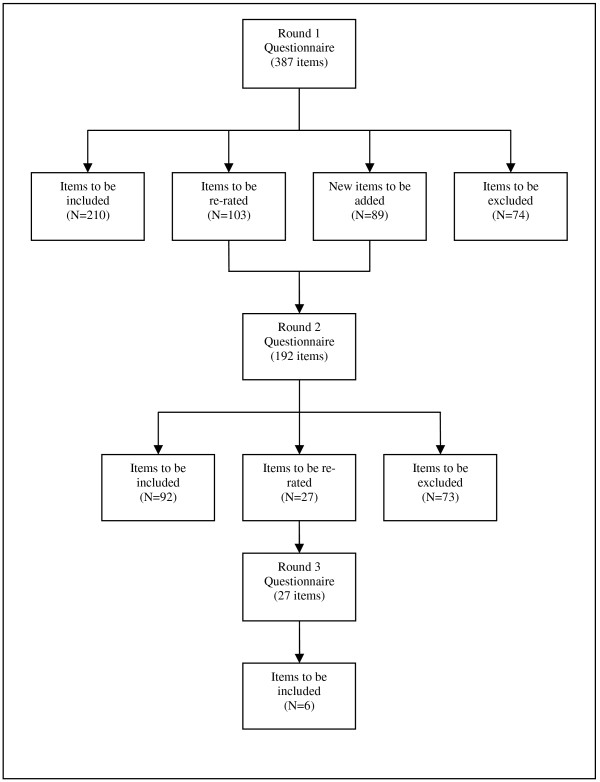
Overview of items included, excluded, created and re-rated in each round of the survey.

See Figure 1 for an overview of the numbers of items that were included, excluded, created and re-rated in each round of the survey. Across three rounds, 308 strategies were rated as essential or important by at least 80% across all three panels. Overall, ratings of whether items were essential or important were similar across the consumer, employer and professional panels, with correlations of r = 0.89 between health professionals and consumers, r = 0.92 between health professionals and employers and r = 0.86 between employers and consumers. However, as might be expected, there were some areas for which views tended to differ. Items that received notably lower ratings from consumers than from employers and health professionals included those relating to the need to remain in work, contact during a sickness absence, monitoring work performance, and for employers to contact or collaborate with treatment providers. Items that received notably higher ratings from consumers than the other panels included those relating to having time off to attend medical appointments, employer-funded counselling services, mediation to manage communication or conflict while absent from work, confidentiality, encouraging support from colleagues after RTW, involving consumers in developing RTW policy, and mental health-related training.

Items that received notably lower ratings from health professionals than consumers and employers included those that related to employer involvement in treatment and relapse prevention. They also gave lower ratings to the statement that the organisation should respond to common mental disorders in the same way that they respond to physical health problems such as back injury, a statement often used to refer to the need to place the same importance on mental health problems as on physical health problems. Items that received higher ratings from health professionals included those relating to employer contact with an employee during sick leave, encouraging the employee to remain in work or to (partially or wholly) RTW before they are completely recovered. They also gave higher ratings to the statement that, with written consent from the employee, the supervisor should also contact the employee’s healthcare provider highlighting any factors that might have a bearing on the employee’s RTW that may be relevant for the healthcare provider to know.

One of the authors (AR) prepared a draft of the guidelines by grouping items of similar content under specific headings. The guidelines retained the original wording of the items as much as possible, whilst remaining easy to read. The draft guidelines were then given to panel members for final comment, feedback and endorsement. No changes were asked for by panel members at this stage.

The final guidelines (see Additional file 1) provide information and advice on how organisations should support those returning to work after common mental disorders. The main points are summarised in Additional file 2. The guidelines cover organisational policy and procedures around RTW, what staff need to know about mental health and RTW, the role of the RTW coordinator, the role of the supervisor, and what the returning employee, colleagues, trade union representatives, and family and friends can do to support RTW. They also provide guidance on what organisations can do to support RTW, including having a RTW plan and providing information/awareness to staff.

## Discussion

The project aimed to identify strategies that could be implemented by organisations supporting those returning to work after common mental disorders. Overall, 308 strategies were endorsed from a comprehensive range of suggestions. The endorsed strategies were written into a guidelines document which is freely available to organisations in order to inform policy and practice.

When responses between panels were compared, health professionals were more likely to rate remaining in work and maintaining contact with employers during absence as essential or important than consumers. This may be due to health professionals’ awareness of some evidence indicating that minimising the duration of sickness absence and supervisory support are linked to improved RTW and health outcomes [28-31]. However, studies exploring employee perspectives in cases of sickness absence due to common mental disorders report that concerns about reduced working capacities, difficulty setting limits in demanding work situations, a high sense of responsibility and a fear of being a burden to an employer acted as barriers to RTW [32,33]. While individual situations obviously vary greatly, and employees themselves report difficulty in estimating the right time to RTW [34], it is likely that RTW outcomes will be improved by employees, employers and health professionals working together to explicitly address these concerns.

Items that received notably lower ratings from employers than consumers and health professionals included those relating to working with trade union representatives, the employee remaining in work and communication about keeping the position open and working with healthcare providers and consumers to develop policy. Employers were also less likely to rate phased RTW as important, which may be of concern due to evidence linking partial RTW with a reduced risk of long-term disability [35]. Items that received higher ratings from employers included those relating to monitoring the working performance and health of employees who have a mental health problem, offering on-the-job support and mentoring schemes to employees returning to work, maintaining contact between the workplace and the employee during absence as well as explaining absence and RTW procedures and discussing treatment issues. While employers have responsibilities in regard to policies and procedures and the health and safety of their employees, monitoring performance is often identified as a sensitive issue for employees and it is likely that RTW outcomes will be improved if this is well handled, often in a clear RTW plan [32,34].

One of the challenges of a project aiming to develop guidelines for organisations as diverse as workplaces is to make them specific enough to be useful while remaining broad enough to be relevant to organisations of various types and sizes. One area that exemplifies this difficulty is that of the role of the RTW coordinator. While large organisations are likely to have a staff member in this role, smaller organisations are not likely to do so. This was reflected in the early rounds of the questionnaire, with respondents commenting that many of the items referring to supervisors should in fact, refer to RTW coordinators. In later survey rounds, we used the term ‘employer representative’ to refer variously to the RTW coordinator, human resources professional or supervisor and asked panellists to re-rate items accordingly.

As mentioned above, the initial questionnaire was developed through a search of available literature. It is notable that there was very little available literature covering a number of areas, including the role of colleagues, family and friends. Employees nominate social support, including social support at work, as important factors in RTW, both during the absence period and after RTW [36]. In the current study, for example, employees gave notably lower ratings than health professionals and employers to the item relating to the need for colleagues to avoid giving advice on dealing with common mental disorders. Further research should explore this aspect of the RTW process.

These guidelines may be compared to those developed in other countries, such as the UK, the Netherlands and Canada. The current guidelines are aimed at supervisors, human resource personnel, RTW coordinators and, in taking a systems approach, are similar to the National Institute for Health and Clinical Excellence (NICE) guidelines developed in the UK, with their focus on identifying a RTW coordinator, developing a consensus about the RTW plan, encouragement of employees to contact a health provider, emphasis on functional capacities, the need for management training and maintaining regular contact with employees [37]. The Canadian best-practice guidelines also take a systems approach and have a focus on organisational policies and practices, effective stakeholder communication, reasonable adjustments and evidence-based treatments [38]. The Dutch guidelines, which have been developed for occupational physicians, have a stronger focus on the individual and on relapse prevention [39].

Limitations of the study include the difficulty in applying many of the recommendations contained in the guidelines in different organisational contexts. Moreover, we aimed to develop guidelines for RTW after high prevalence mental disorders and did not attempt to differentiate between disorders based on severity. Further work may need to explore how guidelines for more severe disorders may differ. However, it is possible that, while implementation of the guidelines may vary according to severity, many of the principles would be the same. Further limitations relate to the online Delphi process, including the possibility that some panel members were asked to advise on issues/questions that were outside their expertise. Some panel members also raised the issue of difficulty in rating some of the items, due to the wide range of organisational environments in which they might apply.

## Conclusions

Developing and building on consensus between employers, employees and health professionals is of critical importance in improving RTW outcomes, as evidence suggests that interventions for common mental disorders that do not consider workplace factors are not likely to show a positive effect on work outcomes [40]. Interventions should therefore be carried out close to the workplace and in collaboration with key stakeholders in order to maximise the chances of success. It is hoped that the development of these guidelines can contribute to this process. Further research is needed to explore how these guidelines might be implemented in a range of organisations. In addition, further research might explore whether the same strategies would be useful for other mental disorders, such as psychosis.

## Competing interests

The authors declare that they have no competing interests.

## Authors’ contributions

NJR and AFJ designed the study with input from EJK and AR. AR completed the literature review, initial survey construction, recruitment of participants, data collection and analysis and prepared drafts of the guidelines. A working group consisting of all authors gathered regularly to give feedback and make improvements on each survey draft and the final guidelines. NJR wrote the first draft of the manuscript with input from AFJ and AR. All authors have contributed to and approved the final manuscript.

## Pre-publication history

The pre-publication history for this paper can be accessed here:

http://www.biomedcentral.com/1471-244X/12/135/prepub

## Supplementary Material

Additional file 1 Helping employees successfully return to work following depression, anxiety or a related mental health problem guidelines for organisations. Click here for file

Additional file 2 Key points for organisations to help employees successfully return to work following a mental health problem. Click here for file
