# Supplementary material for: Development of guidelines to assist organisations to support employees returning to work after an episode of anxiety, depression or a related disorder: a Delphi consensus study with Australian professionals and consumers
Source: BMC Psychiatry. 2012 Sep 3;12:135. doi: 10.1186/1471-244X-12-135 (PMC3551742; doi:10.1186/1471-244X-12-135)
Supplement: Additional file 1 — Helping employees successfully return to work following depression, anxiety or a related mental health problem guidelines for organisations. [file 1471-244X-12-135-S1.pdf]

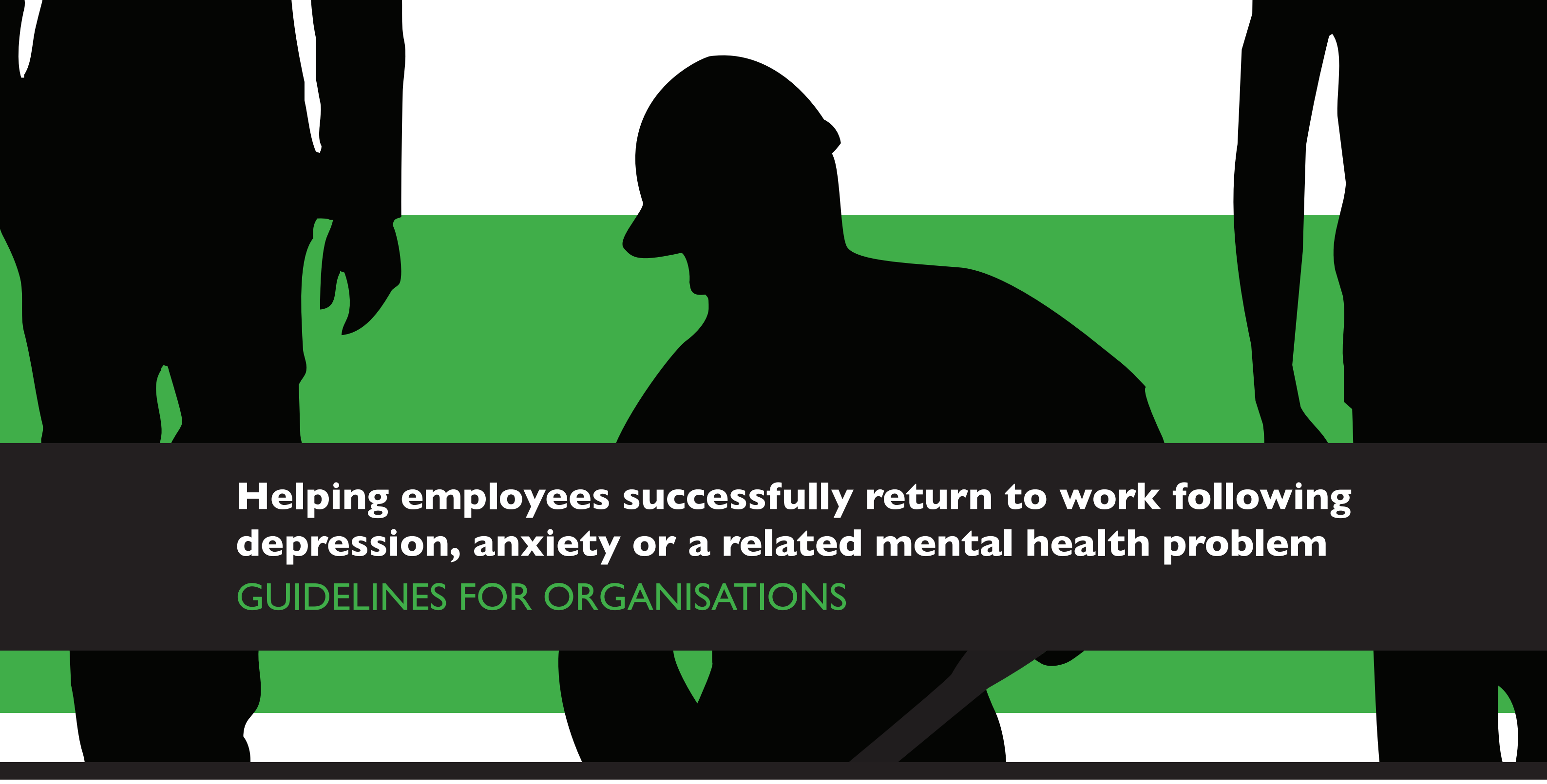

Helping employees successfully return to work following depression, anxiety or a related mental health problem

GUIDELINES FOR ORGANISATIONS

These guidelines consist of actions organisations can take to facilitate return to work for employees following an episode of depression, anxiety or a related disorder. They were produced using the Delphi method, which is a systematic way of assessing the consensus of a panel of experts. The actions have been rated as important or essential by expert panels of consumers, employers and health professionals. It is hoped that the guidelines will be used to improve the practices of organisations as they support those returning to work after mental health problems.

Organisational policy and procedures around return to work

As part of a broader health and wellbeing policy, the organisation should have a specific policy around return to work for employees with a mental health problem. This return to work policy should be formalised and written in plain language, to ensure that it is clear who is responsible for carrying out any actions or procedures.

The organisation should promote awareness and a clear understanding of the policy to all employees, and should ensure that it is implemented, supported and promoted by all stakeholders. The organisation should also ensure that everyone understands their responsibilities relating to return to work, that everyone has the skills and knowledge to put their responsibilities into practice, and that the policy is implemented consistently for all affected employees.

An ideal return-to-work policy should include at least the following:

- **a commitment to helping employees return to work** after sick leave due to a mental health problem, and encouraging their return to work through adjustments rather than prolonging sickness absence 'to play it safe'
- **expectations, roles and responsibilities of all parties** involved in the return-to-work process
- **what should happen when someone discloses** a mental health problem, with a commitment to ensuring that employees who have experienced a mental health problem are treated fairly, equally and consistently
- **how supervisors should seek advice** regarding an employee's mental health problem, the actions they should take, and when and how this action will be supported by the organisation
- **sources of advice within the organisation** on what can be done to help an employee's return to work and continued employment
- **the reasonable adjustments that can be made to retain an employee** who has developed a mental health problem so they are not put at a disadvantage in their job, including provision of time off to attend medical appointments
- **procedures for keeping in contact with staff on sick leave**, including when and how employees should notify absence and what is expected from the employee while on sick leave
- **provision for return-to-work plans** with agreement of everyone affected
- **defining responsibilities for putting the return-to-work plan into action** and reviewing its progress, including arrangements for return-to-work interviews
- **links with other key policies**, such as human resources, health and safety, equal opportunity etc., and company employee benefit schemes

Feedback on the return-to-work policies and procedures should be invited from employees and from employee representatives, with the content reviewed regularly.

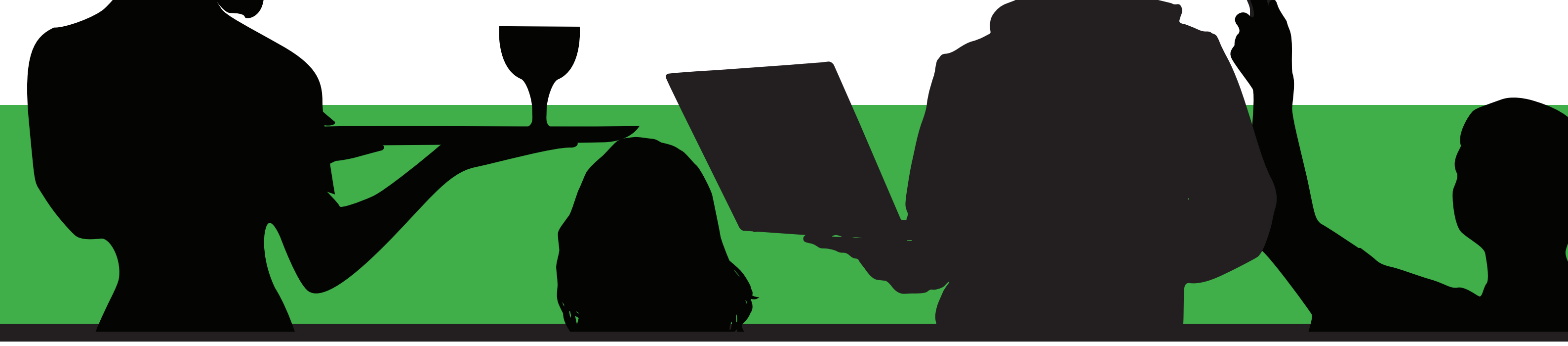

What organisations can do

The work environment

The organisation should foster a supportive work environment that is conducive to good mental health and the enhancement of mental wellbeing. This can be achieved by:

- encouraging staff to discuss stress and seek support when experiencing mental health problems
- fostering a culture in which disclosure of mental health problems is accepted
- taking action to reduce the stigma surrounding mental health problems
- adopting a positive attitude towards those recovering from mental health problems

The organisation should be committed to reintegrating all workers with a mental health problem and should make this known to both employees and supervisors.

Mental health training should be provided for supervisors and colleagues to ensure a supportive work environment and decrease stigma surrounding mental health problems, while providing further training for supervisors to enable them to support employees with a mental health problem to remain in or return to work.

Responsibilities towards employees with a mental health problem

When an employee discloses to a supervisor or other organisational representative that they have a mental health problem, confidentiality should be respected unless there is an immediate danger to the person or to others in withholding that information. To reduce misunderstandings which could lead to fears of discrimination, the organisation should make clear the purpose for which they request or require information about an employee's mental health problem. The organisation should ensure that the employee knows who they can talk to other than their supervisor about their mental health problems (e.g. a human resources professional, occupational health provider or trade union representative).

The organisation should provide information to employees with a mental health problem on taking sick leave due to a mental health problem or returning to work after a mental health problem. This should include information on the positive role of work in recovery from a mental health problem. The organisation should never assume that an employee diagnosed with a mental health problem needs to take leave to recover and should support employees with a mental health problem to stay in work and prevent long-term sickness absence.

The organisation should encourage employees with a mental health problem to obtain treatment. Where practicable, the organisation should offer their employees the support of occupational health advisers or counsellors. The costs of mental health treatment may be offset by gains made in reduced absenteeism and improved productivity at work.

Managing absence

The organisation should make sure that the employee understands their responsibility to keep it informed of the reasons why they are absent from work and, when known, how long the absence is likely to last. The organisation should also provide advice to supervisors on how to appropriately contact an employee who is on leave because of a mental health problem.

If the supervisor has contributed, or is alleged to have contributed, to the employee's mental health problem, the organisation should delegate someone else to maintain contact with the absent employee. If the employee's mental health problem is related to conflicts at work, the organisation should engage the services of qualified workplace mediators.

Managing return to work

The organisation should have a coordinator who facilitates employees' return to work. This person could, for example, be a human resources professional. The coordinator needs to become familiar with the employee's work environment and job content, be able to communicate and negotiate with staff at all levels, and be sensitive to the needs of the employee concerned, including any disability issues. The return-to-work coordinator should also check that the supervisor of the employee has been informed about the return-to-work process, and agrees with it and its possible financial consequences.

The organisation should provide a clear set of behaviours that can be referred to when guiding supervisors on how to manage an employee's return to work, as well as provide additional support, guidance and training to supervisors when they are preparing to manage an employee returning to work from sick leave due to a mental health problem.

A return-to-work assessment of both the job and the employee's mental health should take place, and the organisation should make sure that the supervisor understands their role, the employee knows what to expect and everyone is clear about who is responsible for action in the return-to-work process. A return to work plan should be developed for the employee. (See Box A)

Box A:

THE RETURN-TO-WORK PLAN

When developing an employee's return-to-work plan, the organisation should ensure that:

- **its approach is fair and consistent, while being flexible with details**
- **its approach is tailored to the individual**
- **it offers a variety of options to the employee for a flexible return to work**
- **it provides the employee with adjustments, flexible working practices or job task modifications to accommodate their capabilities**
- **these adjustments are carefully monitored and evaluated, with improvements made where appropriate**
- **it informs employees of disability management initiatives so that they have a greater awareness of roles and resources for making adjustments**
- **those who need to know are informed about reasonable adjustment arrangements made for employees**

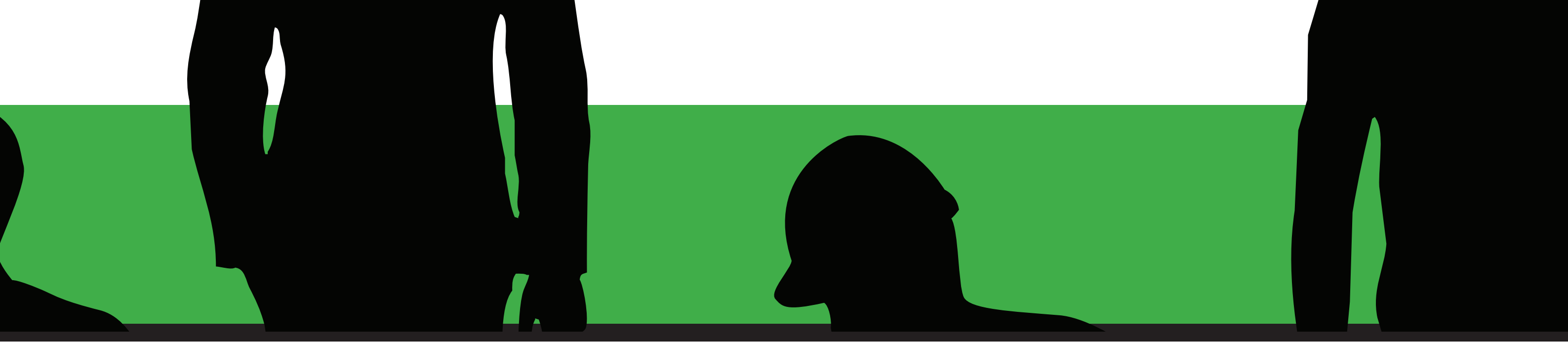

What staff need to know

The organisation should have procedures for making the supervisor, the employee and colleagues aware of the following:

- what a mental health problem is
- how common mental health problems are
- the types of mental health problems
- the warning signs and symptoms of mental health problems
- the causes of mental health problems
- the work-related causes of mental health problems
- the importance of early identification and intervention for preventing or limiting relapse in an employee with a mental health problem
- the things they may notice which might indicate that an employee has a mental health problem, such as effects on attendance, completing work tasks and displaying unusual behaviours
- the benefits for employees of disclosing their mental health problem to the organisation (e.g. to allow access to supports)
- the fears employees may have about disclosing their mental health problem (e.g. stigma from others and not wanting to identify as 'crazy')
- the impact of the symptoms of mental health problems on the skills necessary for work, such as problems with concentration, memory, decision making and motivation
- that the level of support needed by employees with a mental health problem will fluctuate, as the symptoms of most mental health problems come and go over time
- how they can reduce stressors that increase employees' risk of relapse of mental health problems
- how they can support employees with a mental health problem in ways that promote recovery
- how to interact with an employee who is in a distressed state
- how to respond in a mental health crisis situation
- the mental health and disability support services available through the organisation and in the community
- that the negative attitudes of others can be a major problem for an employee with a mental health problem
- the myths surrounding health problems which lead to stigma and limit the potential productivity of employees affected by mental health problems
- the relevant laws and organisation policies that affect interaction with employees with a mental health problem (e.g. Disability Discrimination Act 1992)
- that it is not necessary to be without symptoms of the mental health problem to function successfully at work
- that symptom improvements and work performance improvements may happen at the same or different rates
- that, despite looking fine, the employee may still be ill
- that most people with a mental health problem who receive treatment respond with improved work performance
- the value of work for health and recovery
- that the employee might be anxious about returning to work

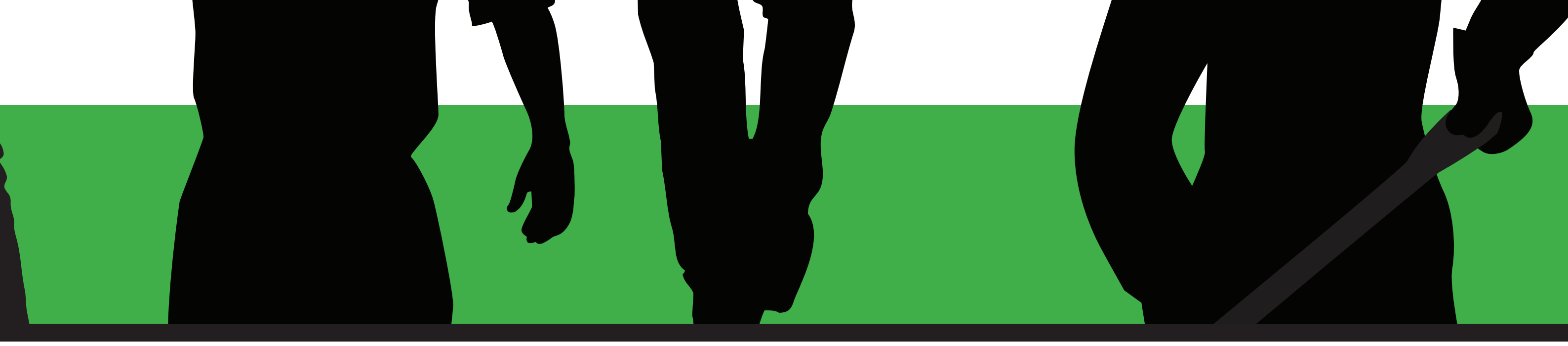

The role of the return-to-work coordinator

Organisations will vary in size and whether or not they have specialist staff to deal with return-to-work issues. In a large organisation, the return-to-work coordinator may be a human resources professional, while in a small organisation it may be the employer or the supervisor. In this section, the 'return-to-work coordinator' refers to any of these people as appropriate.

Managing mental health problems in the workplace

The return-to-work coordinator should be someone who is acceptable to the employee. They should not see everything that the employee says or does as linked to their mental health problem, and should avoid making assumptions about the employee's medical circumstances or what the employee finds stressful or demanding. If the employee is having great difficulty functioning at work, the return-to-work coordinator should discuss appropriate leave arrangements with the employee.

Once an employee has disclosed their mental health problem, it is vital that the return-to-work coordinator discusses and agrees with them exactly who else, if anyone, might need to know, and what information they need to be provided with. The return-to-work coordinator should also make the employee aware that anything they discuss with them about their mental health problem will be kept confidential, unless there is an immediate danger to the person or to others in withholding that information.

Contact during the employee's absence

Where an employee is on sick leave due to a mental health problem, the return-to-work coordinator should maintain an appropriate level of regular contact with the employee. What is appropriate will depend upon the circumstances, and an employer should avoid putting pressure on an employee. The return-to-work coordinator should negotiate and develop a plan for how they will keep in touch with the employee and how often. The return-to-work coordinator should ask the employee who they would prefer to have as their main contact.

When keeping in contact with the employee, all communication between the return-to-work coordinator and the employee should come from a position of care and concern for the employee. The return-to-work coordinator should let the employee know that they are not checking up on them, just keeping them up to date, and should avoid mentioning that colleagues or teammates are under pressure or that work is piling up. The return-to-work coordinator should keep a record of contact made with the employee while on sick leave.

When contacting the employee, the return-to-work coordinator should use these contacts to:

- find out what help and support the organisation can provide
- explain the return-to-work process to the employee
- discuss any work-based issues that would assist them to feel confident and comfortable about returning to work
- discuss reasonable adjustments to assist them upon their return
- reassure the employee about practical issues such as their job security and deal with financial worries
- encourage absent employees to talk to their own doctor, or other healthcare adviser, about what they may be able to do as they make progress or adjust to their condition
- ensure that the employee is aware of the sickness absence and disability policies

At the end of each conversation with the employee, the return-to-work coordinator should agree on when the next follow-up contact will be.

If the employee is too unwell to be contacted directly, the return-to-work coordinator should explore if there is someone else, such as a family member or friend, who can keep in touch on their behalf until the employee is well enough for direct contact.

Managing return to work

The return-to-work coordinator should consider the approach to managing return to work that they would take if an employee had a physical illness, as many of the principles will be the same for a mental health problem. They should proactively seek support and resources for managing an employee's return to work from relevant sources (e.g. human resources and occupational health professionals, beyondblue, SANE, Chamber of Commerce, unions).

With written consent from the employee, the return-to-work coordinator should also contact the employee's healthcare provider. This allows for a two-way flow of information and increases the coordination of support for return to work. The return-to-work coordinator can highlight any factors that might have a bearing on the employee's return to work that may be relevant for the healthcare provider to know. In addition, the healthcare provider can clarify for the return-to-work coordinator any advice they may have to assist in an employee's return to work. However, the return-to-work coordinator should be aware that if the employee does not wish them to contact their healthcare provider that is their right.

If the return-to-work coordinator is concerned that an employee is not yet ready to return to work, they should ask the employee to provide a report from a healthcare provider stating that they are ready to work. When the employee is back at work, the return-to-work coordinator should conduct a return-to-work interview or discussion (see Box B).

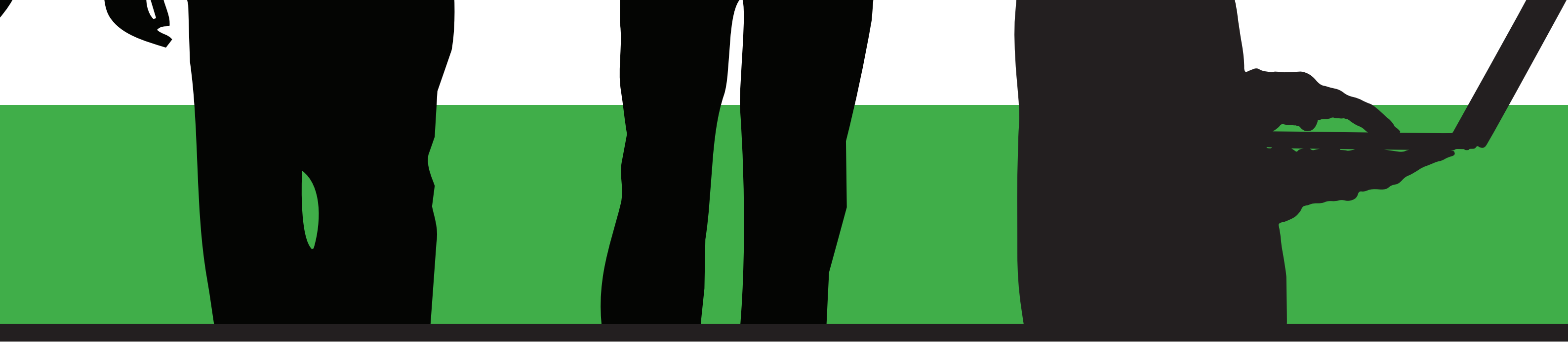

Box B:

WHAT RETURN-TO-WORK DISCUSSIONS SHOULD COVER

During return-to-work discussions, the return-to-work coordinator should:

- **discuss the return-to-work expectations of the employee by clearly explaining policies regarding sick days, time off and other matters related to employee well-being**
- **explain any changes in the employee's role, responsibilities and work practices that have occurred during their absence**
- **focus on the employee's abilities and their capacity to carry out their work, rather than on their limitations**
- **focus their discussions with the employee on the problems they experience in the workplace and what actions can be taken to address them, rather than on details of the mental health problem**
- **find out how the employee's symptoms and treatment impact on their work and think about how this impact can be reduced**
- **discuss with the employee whether any adjustments need to be made to ease their return to work, while being honest about the adjustments they can make and those they can't by explaining that some organisational factors are out of their control**
- **make sure the employee understands the effect of any adjustments on their pay and other entitlements (e.g. effects of reduced hours or alternative work)**
- **discuss the employee's right to confidentiality and reach an agreement on when it would be appropriate to contact a doctor or family member if they become unwell at work**

The role of supervisors

Information and training

The organisation should provide information to supervisors on how they can support an employee who is off work or returning to work after sick leave due to a mental health problem, including:

- **their responsibilities for managing** the attendance and return to work of employees with a mental health problem
- the importance of **good people management skills**, including effective communication, rather than being knowledgeable about the employee's mental health problem
- the organisation's **policy on return to work** and how to put this into practice
- that **successful return to work** is linked to support from managers, the degree of control over work flexibility, the demands placed on them at work, the clarity of their role within the organisation, and their relationships at work
- the important **role the employee plays** in their own return-to-work process
- what organisational resources might be available for **workplace adjustments**
- how to introduce workplace adjustments and **monitor the return-to-work process**
- the **factors that make it difficult to return to work** and how they can minimise their impact
- what **entitlements** are available (e.g. family, sick and annual leave) for employees with a mental health problem
- their **legal responsibilities**, such as those under the Disability Discrimination Act
- understanding that it is their role to **assist the employee to get the help they may need,** but not to diagnose mental health problems or to provide counselling
- recognising the **limitations of a supervisor's responsibility** towards an employee with a mental health problem

Supervisors also need to know how to communicate with employees on sick leave due to a mental health problem. Good communication requires:

- being sensitive to and understanding the individual and their context
- managing the expectations of an employee returning to work
- knowing how to have sensitive conversations with employees, including how to handle those that do not go as planned

Mental health training for supervisors should include opportunities to practice new skills during the training, and remind supervisors that they too are employees who can expect help to return to work if they experience a mental health problem.

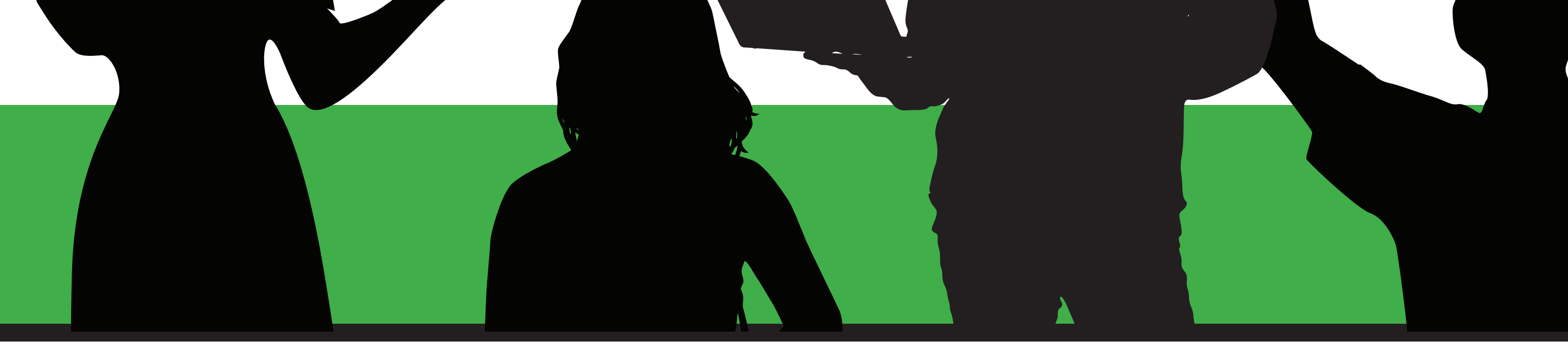

Communicating about mental health problems in the workplace

The supervisor should demonstrate and encourage awareness, understanding and openness in relation to the issues of stress and mental health problems in the workplace. They should acknowledge the impact the employee's mental health problem has on the employee, while making the individual feel that they are a valued employee in the organization.

Managing return to work

If the supervisor is also the return-to-work coordinator, they should refer to the recommendations in the section 'The role of the return-to-work coordinator'. Below are guidelines for supervisors irrespective of whether or not they are the return-to-work coordinator.

During return-to-work discussions, the supervisor should:

- discuss signs and symptoms of any relapse with the employee and ask how they feel these are best dealt with
- sensitively deliver unwelcome news about their absence or position in the workplace
- not make promises to the employee that they cannot keep

The supervisor should also be aware that if the employee does not wish them to contact their healthcare provider that is their right.

Reasonable adjustments

The supervisor should make reasonable adjustments for the employee in the workplace that remove any barriers that prevent an employee from fulfilling their role to the best of their ability. The supervisor should examine the employee's work role to determine whether there are any factors in the workplace that may have contributed to their mental health problem. This includes thinking about how the workplace or the person's workload may be contributing to the problem and considering if any changes can be made.

When making reasonable adjustments, the supervisor should be flexible and treat each case individually, but on a fair and consistent basis. They should avoid making stereotypical assumptions about the capabilities of employees with a mental health problem. The supervisor should also make sure that any side effects of treatment the employee experiences are considered against their job requirements. This is particularly crucial in jobs where there are health and safety risks. These adjustments should be regularly reviewed by the supervisor.

The supervisor should support the employee to access treatments by allowing the person time off work to attend appointments and should investigate other workplace supports that may be available to the employee, such as an Employee Assistance Program (EAP), rehabilitation services or a local employment service.

Managing the returning workplace environment

The supervisor should be realistic about workloads and be aware that some people will wish to prove themselves and may offer to take on too much. Instead, the supervisor should set achievable goals that make the employee feel that they are making progress. To make the employee's first weeks back as low-stress as possible, the supervisor should make sure the employee doesn't return to an impossible in-tray, thousands of emails or a usurped workspace. The supervisor should watch out for hostile reactions to the employee's return to work and promptly stamp out any hurtful gossip or bullying.

The supervisor should take the time to have frequent informal chats so there is an opportunity to discuss progress and problems without a formal (and possibly intimidating) session. However, the supervisor should ensure that the employee does not feel that their work or behaviour is being overly monitored or scrutinised, or that they feel like a nuisance for creating extra work for the supervisor. The supervisor should ensure that any concerns raised by the employee are investigated and dealt with quickly.

The supervisor should continue to support the employee's return to work well beyond the initial return. However, the supervisor should avoid fostering a dependent relationship with an employee with a mental health problem through providing excessive support. They should reassure the employee that they understand medical and personal boundaries and will respect them.

Supervisors should work with healthcare and rehabilitation providers to support employees with mental health problems, and identify physical and psychological factors in the work environment that can be adjusted to accommodate an early, safe and sustainable return to work.

If there are signs of a relapse, the supervisor should review options for making further adjustments and talk realistically with the employee about the best way to move forward. In the case of relapse after return to work, the supervisor should not make any assumptions about whether or not the employee is able to continue working.

If a disciplinary process is required for the employee, the supervisor should have a range of options available for this process, such as counselling, referral to an occupational health specialist etc., as well as the more usual system of warnings.

If the organisation does not have a return-to-work policy, the supervisor should suggest that they have one.

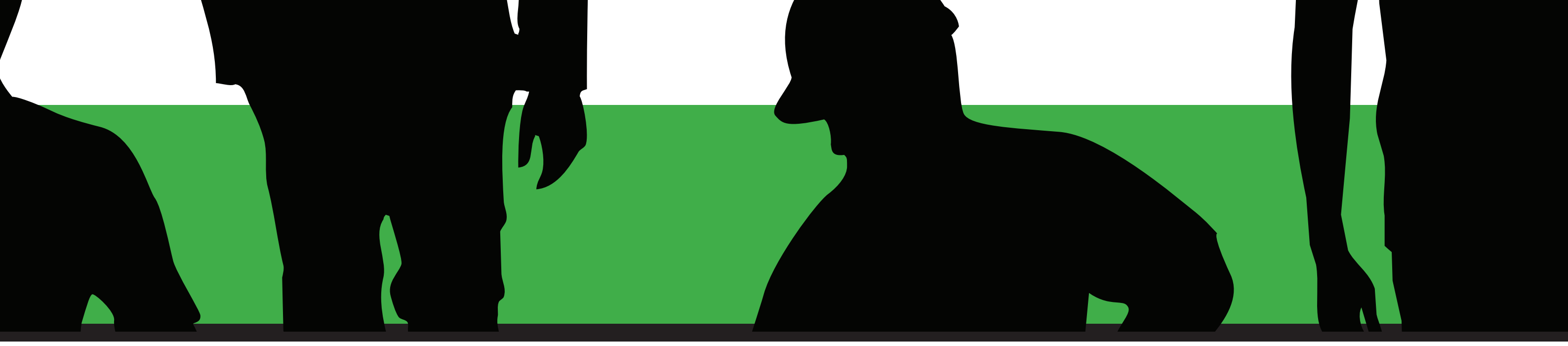

Having a return-to-work plan

Development

A clear written return-to-work plan should be developed by the return-to-work coordinator in discussion with the employee (see Box C). The organisation should ensure the employee is actively involved in the development of the return-to-work plan, and that it is agreed to by everyone affected by the plan.

The return-to-work plan should be flexible and adjustable to allow for changes in the employee's mental health or workplace circumstances, and should last for a sufficient time period to allow the employee to recover. When agreeing on a return-to-work plan, the supervisor should always be clear on the duration of any amended duties or supports. In most cases, these measures will be temporary.

The return-to-work plan should be constantly monitored to ensure that tasks and hours remain appropriate and sufficient supports and resources remain available. The supervisor should take steps to keep everyone informed and make sure the plan is respected.

Box C:

WHAT A RETURN-TO-WORK PLAN SHOULD INCLUDE

- **the approximate date of the employee's return to work**
- **the time period of the plan**
- **the roles and responsibilities of all involved in the plan**
- **a description of suitable duties for the employee**
- **any reasonable adjustments that assist the employee to remain at work and remain well (e.g. return to a modified work role or system, or alternative working hours, whether on a temporary or permanent basis)**
- **information about any impact of adjustments on terms and conditions of employment (i.e. on leave, superannuation, other employment benefits)**
- **any advice received from healthcare providers, human resources etc.**
- **strategies to handle stress, particularly around workplace activities that may trigger this for the employee, such as short deadlines, early meetings or workplace conflict**
- **the process of ensuring the plan is put into practice**
- **the process by which the plan will be reviewed, including review dates and by whom**
- **signatures of agreement – employee, supervisor etc.**

What the returning employee can do

The employee should talk to their supervisor and raise any concerns they might have about their return to work. They should also negotiate with their supervisor about reasonable adjustments to assist with their mental health problem.

The employee should learn the symptoms and triggers of their mental health problem. They should understand that mental health problems are sometimes unpredictable, and that their impact on both cognitive and interpersonal functioning may make work a challenge. The employee should identify perceived barriers and prioritise solutions for a safe and early return to work.

The employee should discuss with their return-to-work coordinator about how to approach their return to work and manage their mental health problem in the workplace. When talking to their return-to-work coordinator about returning to work, the employee should discuss:

- what their tasks and responsibilities will be
- any work activities that may trigger stress and what helps to reduce or manage this stress
- how much they can disclose to work colleagues
- barriers to a safe and early return to work
- any specific needs they have (e.g. time off to attend appointments, inability to do the job in the same way as before becoming unwell)

The employee should discuss with a healthcare professional about how to approach their return to work and manage their mental health problem in the workplace. This should include discussion of any adjustments to their work that may be needed on a temporary or permanent basis and how any medication side effects may affect their work. During a return-to-work assessment by a healthcare professional, the employee should be aware that it is their responsibility to report any participation and activity limitations that are a result of their mental health problem and which may affect their work. The employee should continue to keep their doctor or treating health professional informed during the return-to-work process.

The employee should ask for support when they need it, whether from family, colleagues or supervisors, and should have an agreed plan with their supervisor to manage the possibility of relapse.

What colleagues can do

Colleagues should welcome back the employee who is returning after sick leave due to a mental health problem and should not avoid talking with the person for fear of saying the wrong thing. Colleagues should be respectful of a fellow employee's confidential mental health history and should not pry for details about it.

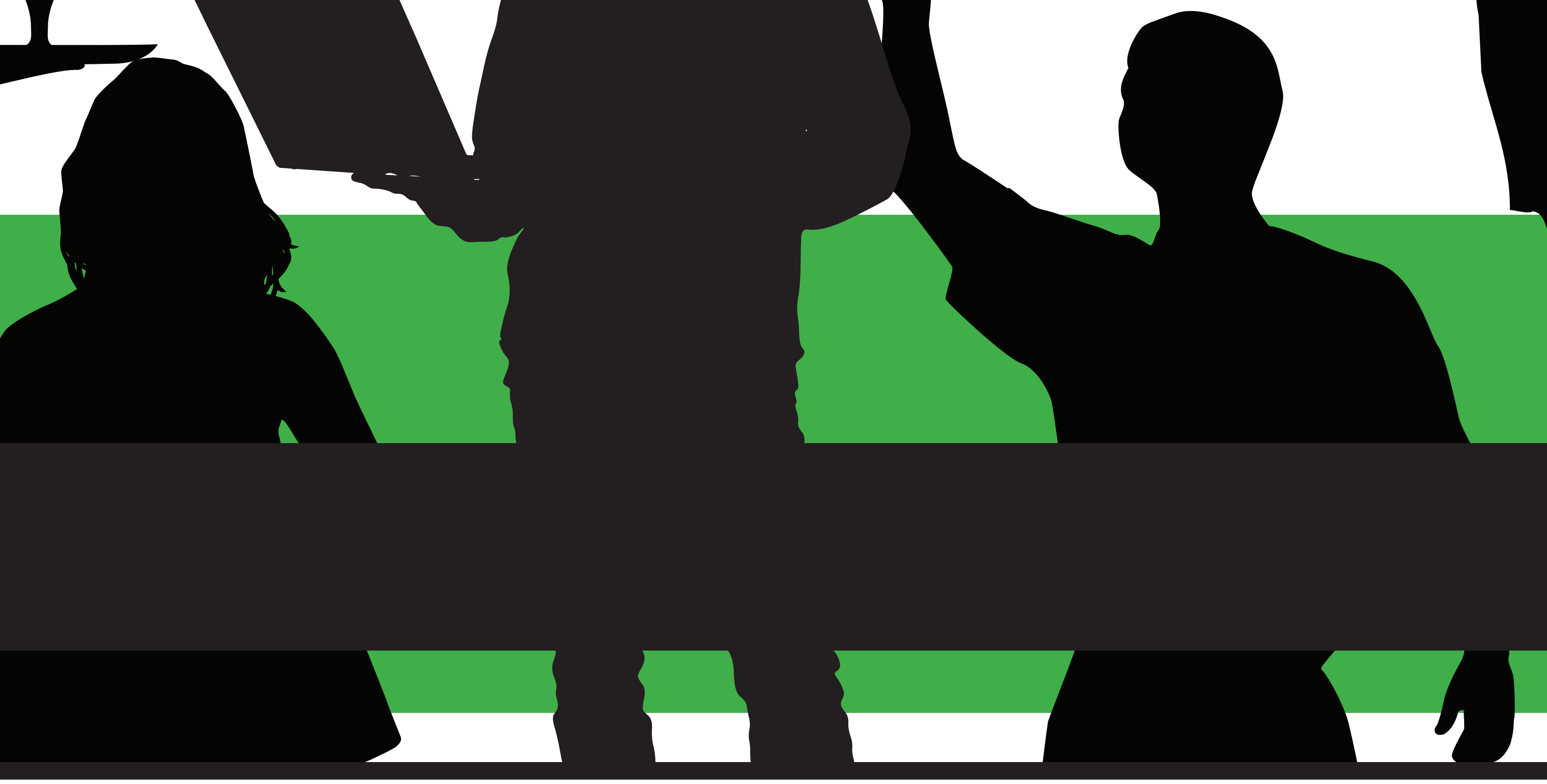

What trade union representatives can do

This section applies only in situations where the employee is a trade union member.

The trade union representative should:

- encourage the employee to co-operate with their employer on a return-to-work plan and use the opportunity offered by a return-to-work interview to ask for help with work-related problems
- reassure the employee that details about their mental health problem cannot be given to others without their consent
- encourage the employee to talk to their doctor about going back to work and about any side effects of medication that may affect their safety or performance at work
- encourage the employee to talk to their doctor about any adjustments to their work that may be needed on a temporary or permanent basis

What family and friends can do

Family and friends should:

- provide practical support to allow the employee to return to work (e.g. childcare, transport, household tasks)
- support the employee in meeting their obligations under the return-to-work plan
- leave the decision about when to return to work to the employee and their health care professional
- not give negative messages to the employee about their ability to return to work
- positively acknowledge success in return to work
- be aware of early signs of relapse and how these may impact on work performance

Family and friends should also be aware that positive emotional support can assist the employee's recovery and return to work, while negative interactions outside the workplace can affect the employee's ability to return to or remain at work.

Although these guidelines are copyright, they can be freely reproduced for non-profit purposes provided the source is acknowledged.

Please cite these guidelines as follows:
Helping employees successfully return to work following depression, anxiety or a related mental health problem: guidelines for organisations
Centre for Youth Mental Health, University of Melbourne; 2011.

Enquiries should be addressed to: Dr Nicola Reavley, Centre for Youth Mental Health, Locked Bag 10, Parkville, VIC 3052, Australia. Email: nreavley@unimelb.edu.au

8671_KJOYH_2012
