# Supplementary material for: Development of guidelines to assist organisations to support employees returning to work after an episode of anxiety, depression or a related disorder: a Delphi consensus study with Australian professionals and consumers
Source: BMC Psychiatry. 2012 Sep 3;12:135. doi: 10.1186/1471-244X-12-135 (PMC3551742; doi:10.1186/1471-244X-12-135)
Supplement: Additional file 2 — Key points for organisations to help employees successfully return to work following a mental health problem. [file 1471-244X-12-135-S2.docx]

## Key points for organisations to help employees successfully return to work following a mental health problem

### Have a policy around return to work

- As part of a broader health and wellbeing policy, the organisation should have a specific policy around return to work for employees with a mental health problem.
- The organisation should promote awareness and a clear understanding of the policy to all employees, and should ensure that it is implemented, supported and promoted by all stakeholders.
- The organisation should also ensure that everyone understands their responsibilities relating to return to work, that everyone has the skills and knowledge to put their responsibilities into practice, and that the policy is implemented consistently for all affected employees.

### Foster an environment that supports mental health

- The organisation should foster a supportive work environment that is conducive to good mental health and the enhancement of mental wellbeing.
- The organisation should be committed to reintegrating all workers with a mental health problem and should make this known to both employees and supervisors.
- Mental health training should be provided for supervisors and colleagues to ensure a supportive work environment and decrease stigma surrounding mental health problems, while providing further training for supervisors to enable them to support employees with a mental health problem to remain in or return to work.
- The organisation should never assume that an employee diagnosed with a mental health problem needs to take leave to recover and should support employees with a mental health problem to stay in work and prevent long-term sickness absence.
- The organisation should encourage employees with a mental health problem to obtain treatment.

### Actively manage absence

- The organisation should maintain an appropriate level of regular contact with the employee.
- The organisation should make sure that the employee understands their responsibility to keep it informed of the reasons why they are absent from work and, when known, how long the absence is likely to last.

### Actively manage return to work

- The organisation should have a coordinator who facilitates employees' return to work. This person should be someone who is acceptable to the employee.
- The return-to-work coordinator should consider the approach to managing return to work that they would take if an employee had a physical illness, as many of the principles will be the same for a mental health problem.
- The return-to-work coordinator should agree with the employee exactly who else, if anyone, might need to know about their mental health problem, and what information they need to be provided with.
- With written consent from the employee, the return-to-work coordinator should also contact the employee's healthcare provider.
- The supervisor should make reasonable adjustments for the employee in the workplace. These should remove any barriers that prevent an employee from fulfilling their role to the best of their ability.
- The supervisor should examine the employee’s work role to determine whether there are any factors in the workplace that may have contributed to their mental health problem. This includes thinking about how the workplace or the person’s workload may be contributing to the problem and considering if any changes can be made.
- A return-to-work assessment of both the job and the employee's mental health should take place.
- If there are signs of a relapse, the supervisor should review options for making further adjustments and talk realistically with the employee about the best way to move forward.

### Develop a return-to-work plan

- A clear written return-to-work plan should be developed by the return-to-work coordinator in discussion with the employee.
- The plan should be agreed to by everyone affected by it, should be flexible and adjustable and should last for a sufficient time period to allow the employee to recover.
- The plan should be monitored to ensure that tasks and hours remain appropriate and sufficient supports and resources are available.

### Involve the employee

The employee should:

- talk to their supervisor and raise any concerns they might have about their return to work.
- learn the symptoms and triggers of their mental health problem.
- identify perceived barriers and prioritise solutions for a safe and early return to work.
- discuss with a healthcare professional about how to approach their return to work and manage their mental health problem in the workplace.
- ask for support when they need it, whether from family, colleagues or supervisors, and should have an agreed plan with their supervisor to manage the possibility of relapse.

### Encourage support from others

- Colleagues should welcome back the employee who is returning after sick leave due to a mental health problem and should not avoid talking with the person for fear of saying the wrong thing.
- Colleagues should be respectful of a fellow employee's confidential mental health history and should not pry for details about it.
- Family and friends should be aware that positive emotional and practical support can assist the employee's recovery and return to work, while negative interactions outside the workplace can affect the employee's ability to return to or remain at work.
